# Health Behaviour in Children and Adolescents with Type 1 Diabetes Compared to a Representative Reference Population

**DOI:** 10.1371/journal.pone.0112083

**Published:** 2014-11-10

**Authors:** Sebastian Kummer, Anna Stahl-Pehe, Katty Castillo, Christina Bächle, Christine Graf, Klaus Straßburger, Burak Salgin, Ertan Mayatepek, Guido Giani, Reinhard W. Holl, Thomas Meissner, Joachim Rosenbauer

**Affiliations:** 1 Department of General Pediatrics, Neonatology and Pediatric Cardiology, University Children's Hospital, Duesseldorf, Germany; 2 Institute for Biometrics and Epidemiology, German Diabetes Center (DDZ), Leibniz Institute for Diabetes Research at Heinrich Heine University, Duesseldorf, Germany; 3 Institute of Movement and Neurosciences, German Sport University, Cologne, Germany; 4 Department of Paediatrics, Cambridge University, Addenbrooke's Hospital, Cambridge, United Kingdom; 5 Institute for Epidemiology and Medical Biometry, University of Ulm, Ulm, Germany; TNO, Netherlands

## Abstract

**Objective:**

We provide a population-based overview of health behaviours of children and adolescents with type 1 diabetes in comparison to the general population, and analyse their relevance for glycaemic control and self-rated health status.

**Methods:**

Data from questionnaires of 11- to 17-year-old children and adolescents with diabetes (n = 629) were compared to a representative sample (n = 6,813).

**Results:**

Children and adolescents with type 1 diabetes had a significantly increased odds of infrequent physical activity (adjusted OR 1.56), short overall duration of physical activity per week (OR 1.55, difference -1.3 hours/week), and high daily computer use (OR 2.51). They had a lower odds of active and passive smoking (OR 0.31 and OR 0.29), and high daily television time (OR 0.68). The odds of an at least good and excellent self-rated health status was increased with intense physical activity, and decreased with active smoking and prolonged daily use of computer and television. Active smoking and prolonged daily use of computer were associated with higher HbA1c.

**Conclusions:**

Children and adolescents with type 1 diabetes showed a different profile of health behaviour. Their overall health may improve if their education stresses specifically frequent physical activity with longer overall duration and less frequent television or computer use.

## Introduction

Long-term prognosis in type 1 diabetes improves with good glycaemic control in childhood and adolescence [1]. This depends not only on adequate insulin substitution, but also on health-relevant behaviours such as physical activity, nutrition, smoking, and psychosocial parameters, which have a significant impact on the quality of glycaemic control. For example, physical activity has the potential to reduce cardiovascular risk, enhance quality of life and improve glucose control in patients with type 1 diabetes [2], [3] as well as in healthy individuals [4]. Also on the tissue level, exercise results in beneficial effects, e.g. on pancreas, cardiovascular system, the kidneys, and skeletal muscle [5] Smoking negatively affects type 1 diabetes management and increases the risk especially for long-term sequelae of diabetes mellitus [6], [7]. It was shown in a clinical sample of youths with type 1 diabetes that daily media consumption time is a significant risk factor for poor glycaemic control [8]. In healthy children and adolescents, computer use and watching television are associated with physical activity, eating behaviour and obesity [9], [10]. However, the impact of these health-relevant activities has not been evaluated in detail for their relevance in patients with type 1 diabetes. To our knowledge, there is no population-based study to date that has compared these health behaviours between children and adolescents with type 1 diabetes and the general population.

Patients with early-onset type 1 diabetes are expected to be at increased risk of late complications compared with patients with later onset [11]. In addition, a doubling of new cases of type 1 diabetes in European children younger than 5 years of age between 2005 and 2020 has been predicted [12]. Associations between physical activity, smoking and media consumption on the one side and glycaemic control and health status on the other side are largely unexplored in this young patient group so far.

Our objective is to provide a population-based overview of health-relevant behaviours and their association with glycaemic control and self-rated health status in children and adolescents with early-onset and long-duration type 1 diabetes in comparison to peers from the general population in Germany.

## Methods

### Data source/study populations

Data sources have been described in detail elsewhere [13]. In brief, young patients with type 1 diabetes mellitus between 11 and 21 years of age and disease onset between age 0 and 4 years in the period from 1993 to 1999 were selected from the nationwide diabetes register (completeness of ascertainment 95%) maintained at the German Diabetes Center, Duesseldorf, Germany. Between September 2009 and December 2010, 2.241 patients and their parents received mailed/paper-based questionnaires, information about the study and a consent form. No financial incentives were paid for participation. Comprehensive questionnaires were obtained from 840 participants and their parents. Participants and non-participants did not differ significantly with respect to sex, but participants were 0.8 years younger, had 0.2 years earlier disease onset and 0.6 years shorter disease duration (each p<0.001). For the analyses, all patients ≥18 years of age were excluded, leaving 629 children and adolescents between 11 and 17 years of age. The overall response rate was 43% in 11- to 13-year-olds and 42% in 14- to 17-year-olds.

As normative data, the Public Use File of the German Health Interview and Examination Survey for Children and Adolescents (KiGGS 2003–2006, Robert-Koch-Institute, Berlin, Germany) was used [14]. Data collection was performed between May 2003 and May 2006 from 17.641 children and adolescents aged between 0–17 years. 6,813 participants were in the age range 11 to 17 years. The response rate was 69% in 11- to 13-year-olds and 63% in 14- to 17-year-olds.

Both studies used identical extensive, standardized, self-administered questionnaires to obtain information from children and adolescents as well as from their parents. The study was approved by the commissioner responsible for data protection and the ethics committee of Düsseldorf University (study number 3254), and was conducted according to the principles expressed in the Declaration of Helsinki. Written and informed consent was obtained from all participating children and adolescents and all parents or respective legal guardians.

### Variables

The children and adolescents answered questions concerning their health behaviour. They were asked how often (never, 1–2 times per month, 1–2 times per week, 3–5 times per week, approximately every day) and how many hours per week in total they were physically active in their leisure time in an intensity to cause sweating or hard breathing (e.g. by sports or bicycling). In addition, they were asked about their own smoking behaviour, and environmental smoking exposure (frequency of active and passive smoking: never, less than once per week, once per week, a number of times per week, every day), and about the average daily use of television and computers (never, about half an hour, about 1–2 hours, about 3–4 hours, more than 4 hours per day). To avoid small numbers of cases in subcategories for some analyses, the cut point for low and high frequency of physical activity was defined at 3 times per week instead of 7 times per week as recommended. For some analyses, duration of physical activity was dichotomized as low (<7 hours/week) and high (≥7 hours/week) according to international recommendations (at least 60 minutes of moderate to vigorous physical activity per day) [15]. The categorization for active and passive smoking was yes/no and >1 time/week/≤1 time/week, respectively. Time spent watching television and using the computer was categorized in ≥3 hours/day and <3 hours/day based on recent recommendations (limit entertainment screen time to <1 to 2 hours per day [16]).

Sociodemographic information was given by the parents. Participants were classified as having a migration background if they had emigrated from another country and at least one of their parents was not born in Germany or was of non-German nationality. Socioeconomic status was defined according to an established composite social status index, which integrated information obtained from the parents' questionnaires on parental level of education, parents' professional status, and household income. Each of the three components was rated with 1–7 points and summed up to a total score (range 3–21 points), which was categorized as low (3–8 points), intermediate (9–14) or high (15–21 points) socioeconomic status. The socioeconomic status is a statistical construct specifically recommended for epidemiological research in Germany [17], [18]. Two regions of residence – East-Germany (former German Democratic Republic including Berlin), and West-Germany (former Federal Republic of Germany) – were differentiated. Weight status was classified as underweight, normal weight, and overweight including obesity using age- and sex-specific German reference data based on the body mass index [19]. Weight and height of the participants were self-reported in the diabetes study, but measured in a standardized way in the KiGGS.

Severe hypoglycaemic events were defined as hypoglycaemia with impaired consciousness and/or convulsion or glucagon treatment, according to 2009 International Society for Pediatric and Adolescent Diabetes (ISPAD) guidelines [20]. Glycaemic control derived from clinical records of a subgroup of 444 youths was classified for some analyses as optimal (HbA1c <7.5%), suboptimal (HbA1c 7.5%–9.0%), and poor (HbA1c >9.0%) according to the ISPAD Guidelines [21]. Self-rated health status had been assessed with the question “How would you describe your health in general?” with a 5-point Likert scale (excellent, good, moderate, poor, very poor).

### Statistical analyses

Percentages or means and standard deviations (SD) are reported as descriptive statistics. A survey weighting factor was used for the KiGGS data to adjust for deviations of demographic characteristics between the sample and the general population in Germany related to age, sex, region and nationality [22]. This approach was necessary to compare data of the diabetes study with nationally representative data. For simple group comparisons of continuous and categorical variables t-test or Wilcoxon-test and χ^2^-test were used, respectively.

Multivariable linear and logistic regression analysis was applied to identify differences between the patient group and the reference group adjusted for confounding factors. Dependent variables were frequency and duration of physical activity, active and passive smoking, time spent watching television, and time using the computer. For each outcome, study group, age, sex, socioeconomic status, migration background, region of residence, and weight status were included as independent variables. To explore differential effects of study groups on outcomes by age and sex, we added terms for study group by age and study group by sex interactions in the regression models. Adjusted differences (β) and odds ratios (OR) with 95% confidence intervals (95% CI) and respective p-values were obtained by applying the SAS SURVEYREG and SURVEYLOGISTIC procedures. Explorative analyses were performed for youths with type 1 diabetes to analyse associations between aspects of health behaviour and severe hypoglycaemic events, glycaemic control, and self-rated health status adjusted for confounders. We applied multivariable ordinal logistic regression (proportional odds model based on cumulative odds) [23] to severe hypoglycaemic events (0, 1≥2 events), glycaemic control (HbA1c <7.5%, 7.5–9.0%,>9.0%) and self-rated health status (ordinal: excellent, good, at most moderate) as ordinal outcomes. A Score test was used to test the proportional odds assumption (identical OR across different cut-offs of the outcome). As the Score test is anticonservative (i.e. the test rejects the proportional odds assumption too readily) [24], the test was performed at an α-level of 0.01. We applied multivariable linear regression to HbA1c as continuous outcome. Health behaviours were modelled as dichotomous and ordinal (linear score term for categories) independent variables. Odds ratios and adjusted differences with 95% confidence intervals (95% CI) and respective p-values were obtained by applying the SAS REG and LOGISTIC procedures. In case of the proportional odds model [23], [24], ORs estimated for severe hypoglycaemic events as ordinal outcome refer to the odds of at least one versus no event and to the odds of at least 2 events versus at most 1 event, ORs estimated for ordinal HbA1c refer to the odds of HbA1c ≥7.5% versus HbA1c <7.5% and to the odds of HbA1c ≥9.0% versus HbA1c <9.0, ORs estimated for self-rated health status refer to the odds of at least good versus at most moderate health status and to the odds of excellent versus at most good health status. All tests were performed two-sided at a 5% significance level (with the exception of the Score test described above). SAS for Windows Version 9.3 (SAS Institute, Cary, North Carolina, USA) was used for all analyses.

## Results

### Description of study populations

A description of the two study populations is given in Table 1. Significant differences between both populations were found for age, socioeconomic status, migration background, region of residence, and weight status. All subsequent between-group analyses were adjusted for these variables. Children and adolescents with type 1 diabetes had a mean age at disease onset of 2.7 years (SD 1.1, range 0.6–4.9 years) and a mean diabetes duration of 12.5 years (SD 1.6, range 10.0–16.5 years). About half of them (48.8%) used continuous subcutaneous insulin infusion (CSII), 43.3% used multiple daily injections (≥4/day, MDI), and 7.9% used conventional therapy (1–3 injections/day, CT). Mean HbA1c among those with available data (n = 444) was 8.4% (SD 1.6); 24.5% of patients had optimal, 50.9% suboptimal, and 24.5% poor glycaemic control. A total of 19.8% of the patients reported excellent and 62.4% good general health. One or at least 2 severe hypoglycaemic events during the last 12 months were reported by 9.3% and 4.2%, respectively.

**Table 1:** Description of the two study populations.

	Diabetes study a	KiGGS b	p-value c
Number of participants	629	6813	
Boys [%]	54.1	51.3	0.189
Age			
Mean (SD) [years]	15.3 (1.7)	14.6 (2.0)	<0.001
11–13 years [%]	24.0	39.6	<0.001
14–17 years [%]	76.0	60.4	
Socioeconomic status			
Low [%]	17.9	27.4	<0.001
Intermediate [%]	48.2	47.2	
High [%]	33.9	25.3	
Migration background [%]	1.8	17.5	<0.001
Region of residence			
West-Germany [%]	86.2	81.4	0.003
East-Germany [%]	13.8	18.6	
Family structure			
Biological parents [%]	79.2	74.6	0.070
Mother and partner/father and partner [%]	9.3	10.6	
Single mother/father [%]	10.4	13.6	
Other (relatives, foster parents, youth institutions) [%]	1.1	1.3	
Weight status			
Underweight [%]	3.3	7.5	<0.001
Normal weight [%]	80.7	74.8	
Overweight (incl. adiposity) [%]	16.0	17.7	

a Percentages and means (SD).

b Weighted percentages and weighted means (SD).

c χ^2^-Test, t-Test.

### Health behaviour in children and adolescents with type 1 diabetes compared to the general population

Table 2 shows the descriptive statistics concerning physical activity, active/passive smoking, and television/computer use. Tables 3 and 4 show the results of the comparison of the two study populations by multivariable logistic and linear regression analyses. The frequency and duration of physical activity differed between the youths with diabetes and the reference group (Tables 2, 3, and 4). The adjusted odds of infrequent physical activity (≤2 times/week) was 56% higher in the total patient group than in the total reference group (adjusted OR 1.56, p<0.001). The crude mean duration of physical activity per week was 5.5 (SD 4.8) hours in the patient group and 7.0 (SD 7.1) hours in the reference group. Adjusted for confounders, children and adolescents with diabetes had on average a 1.26 hours (p<0.001) shorter weekly duration of physical activity than the reference group (Table 4). Concordantly, the adjusted odds for shorter physical activity (<7 hours/week) was increased in the physically active patient group compared to the physically active reference group (OR 1.55, p<0.001) (Table 3). Compared to the general population, children and adolescents with type 1 diabetes mellitus had a lower odds for active smoking (OR 0.31, p<0.001) and passive smoking (OR 0.29, p<0.001). Active smoking was virtually not reported in individuals 11 to 13 years of age. The odds of high daily television consumption (≥3 hours/day) was lower in the patient group than in the reference group (OR 0.68, p = 0.002). In contrast, the odds for youths with type 1 diabetes to have ≥3 hours of daily computer use was higher than in the reference group (OR 2.51, p<0.001).

**Table 2:** Prevalence of health-related behaviours in children and adolescents with type 1 diabetes and KiGGS participants.

Health-related behaviour	Diabetes study [%] a	KiGGS [%] b	p-value c
**Frequency of physical activity**			
Never	6.4	10.2	<0.001
1–2 times/month	10.1	5.4	
1–2 times/week	39.6	29.9	
3–5 times/week	32.4	31.6	
Approximately every day	11.5	22.9	
**Duration of physical activity in physically active youths**			
Mean (SD) hours/week	5.5 (4.8)	7.0 (7.1)	<0.001
>0–<2 hours/week	11.1	7.0	<0.001
≥2–<5 hours/week	41.6	37.7	
≥5–<7 hours/week	19.3	18.6	
≥7–<10 hours/week	13.6	13.5	
≥10 hours/week	14.4	23.2	
**Active smoking**			
No	89.4	79.6	<0.001
Yes	10.6	20.4	
**Passive smoking**			
Never	26.8	14.0	<0.001
Once a week or less often	52.9	45.2	
Several times a week or daily	20.3	40.9	
**Television**			
0 hours/day	5.0	4.1	<0.001
0.5 hours/day	27.8	21.4	
1–2 hours/day	51.7	51.7	
3–4 hours/day	11.3	17.2	
≥4 hours/day	4.2	5.6	
**Computer**			
0 hours/day	6.6	24.0	<0.001
0.5 hours/day	27.9	35.4	
1–2 hours/day	39.5	28.9	
3–4 hours/day	16.0	7.6	
≥4 hours/day	10.0	4.1	

a Percentages and means (SD).

b Weighted percentages and weighted means (SD).

c χ^2^-Test, Wilcoxon-Test.

**Table 3:** Odds ratios for health-related behaviours of children and adolescents with type 1 diabetes compared to KiGGS participants.

Health-related behaviour	Total groups	Girls	Boys		12 years	16 years	
	OR (95% CI) a	OR (95% CI) b	OR (95% CI) b	P^ d^	OR (95% CI) b	OR (95% CI) b	ΔOR per year (95% CI) b
	P c	P c	P c		P c	P c	P e
**Frequency of physical activity**							
≤2 times/week vs. ≥3 times/week	1.56 (1.30; 1.88)	1.36 (1.04; 1.79)	1.75 (1.36; 2.25)	0.172	1.55 (1.07; 2.25)	1.54 (1.26; 1.88)	1.00 (0.90; 1.10)
	<0.001	0.026	<0.001		0.020	<0.001	0.959
**Duration of physical activity** f							
>0–<7 hours/week vs. ≥7 hours/week	1.55 (1.26; 1.91)	1.33 (0.94; 1.87)	1.57 (1.19; 2.05)	0.438	1.25 (0.83; 1.90)	1.59 (1.26; 2.01)	1.06 (0.95; 1.19)
	<0.001	0.105	0.001		0.283	<0.001	0.313
**Active smoking**							
Yes vs. no	0.31 (0.23; 0.42)	0.24 (0.14; 0.42)	0.33 (0.19; 0.55)	0.320	0.25 (0.11; 0.59)	0.30 (0.22; 0.41)	1.05 (0.87; 1.27)
	<0.001	<0.001	<0.001		0.002	<0.001	0.626
**Passive smoking**							
>1 time/week vs. ≤1 time/week	0.29 (0.23; 0.36)	0.37 (0.26; 0.52)	0.24 (0.17; 0.34)	0.061	0.32 (0.19; 0.55)	0.28 (0.22; 0.36)	0.97 (0.85; 1.11)
	<0.001	<0.001	<0.001		<0.001	<0.001	0.641
**Television**							
≥3 hours/day vs. ≤2 hours/day	0.68 (0.54; 0.87)	0.60 (0.42; 0.86)	0.71 (0.48; 1.04)	0.503	0.59 (0.34; 1.002)	0.70 (0.54; 0.90)	1.04 (0.91; 1.20)
	0.002	0.006	0.075		0.051	0.006	0.539
**Computer**							
≥3 hours/day vs. ≤2 hours/day	2.51 (2.01; 3.13)	3.89 (2.71; 5.59)	1.51 (1.09; 2.09)	<0.001	1.80 (1.09; 2.96)	2.93 (2.33; 3.69)	1.13 (0.998; 1.28)
	<0.001	<0.001	0.014		0.021	<0.001	0.055

a Estimated from separate logistic regression models including study group, age, sex, migration background, socio-economic status, region of residence, and weight status.

b Estimated from separate logistic regression model including study group, age, sex, terms for study group by sex and study group by age interactions, migration background, socio-economic status, region of residence, and weight status.

c P-value (Wald-χ^2^-Test); ^d^ P-value (Wald-χ^2^-Test) for comparison sex-specific odds ratios (corresponding to testing of study group by sex interaction).

e P-value (Wald-χ^2^-Test) for testing ΔOR per year (relative change in OR per year, corresponding to testing of study group by age interaction).

f Physically inactive youths were excluded.

**Table 4:** Duration of weekly physical activity of children and adolescents with type 1 diabetes compared to KiGGS participants.

Health-related behaviour	Total groups	Girls	Boys		12 years	16 years	
	ß (95% CI) a	ß (95% CI)^.b^	ß (95% CI) b	P d	ß (95% CI) b	ß (95% CI) b	Δß per year (95% CI) b
	P c	P c	P c		P c	P c	P e
**Difference in duration of weekly physical activity **[hours/week] f	1.26 (−1.70; −0.82)	−0.71 (−1.23; −0.20)	−1.78 (−2.43; −1.13)	0.012	−1.36 (−2.23; −0.50)	−1.17 (−1.72; −0.63)	0.05 (−0.24; 0.33)
	<0.001	0.007	<0.001		0.002	<0.001	0.740

a Estimated from linear regression model including study group, age, sex, migration background, socio-economic status, region of residence, and weight status.

b Estimated from linear regression model including study group, age, sex, terms for study group by sex and study group by age interactions, migration background, socio-economic status, region of residence, and weight status.

c P-value (Wald-χ^2^-Test).

d P-value (Wald-χ^2^-Test) for comparison sex-specific difference (corresponding to testing of study group by sex interaction).

e P-value (Wald-χ^2^-Test) for testing Δß per year (change in ß per year, corresponding to testing of study group by age interaction).

f Physically inactive youths were excluded.

Results of the regression models including terms for study group by sex and study group by age interactions showed that the adjusted ORs for health-related behaviours of children and adolescents with type 1 diabetes compared to KiGGS participants were similar among boys and girls (p>0.05 for testing study group by sex interaction) and independent of age (p>0.05 for testing study group by age interaction). Sex-specific differences between the patient group and the general population were only observed for computer use (OR significantly higher in girls than in boys: 3.89 vs. 1.51, p<0.001) (Table 3), and the adjusted difference in the duration of weekly physical activity (difference smaller in girls than in boys: −0.71 vs −1.78, p = 0.012) (Table 4).

### Associations between health behaviours and hypoglycaemic events, glycaemic control, and self-rated general health status in children and adolescents with type 1 diabetes mellitus

The proportional odds assumption was not rejected in any of the ordinal logistic regression models (Score test p≥0.02), with the exception of the model with hypoglycaemic events as outcome and modelling active smoking as dichotomous independent variable (Score test p = 0.006) (Table 5).

**Table 5:** Prevalence of outcomes in children and adolescents with type 1 diabetes dependent on health-related behaviours and respective odds ratios.

	Outcome							
Health-related behaviour (independent variable)	Severe hypoglycaemic events during past 12 months (ordinal: 0,1, ≥2)		HbA1c (ordinal: <7.5, 7.5–9.0,>9.0)		HbA1c (continuous)		Self-rated health status (ordinal: at most moderate, good, excellent)	
	[%] a	OR (95% CI) c	[%] a	OR (95% CI) c	Mean (SD) e	ß (95% CI) g	[%] a	OR (95% CI) c
	p b	p d	p b	p d	p f	p h	p b	p d
**Frequency of physical activity**		0.650 i		0.822 i				0.085 i
≤2 times/week	11.0	1	24.2	1	8.47 (1.60)	0	14.7	1
≥3 times/week	16.4	1.75 (1.07; 2.86)	26.8	1.20 (0.82; 1.74)	8.44 (1.51)	0.17 (−0.12; 0.46)	26.4	1.70 (1.21; 2.34)
	0.050	0.026	0.468	0.351	0.876	0.259	<0.001	0.002
Ordinal coding for categories (never, 1–2 times/month, 1–2 times/week, 3–5 times/week, approximately every day)		0.460 i		0.825 i		0.05 (−0.12; 0.21)		0.023 i
		1.29 (1.01; 1.66)		1.08 (0.89; 1.30)		0.583		1.30 (1.10; 1.53)
		0.045		0.441				0.002
**Duration of physical activity j**		0.577 i		0.651 i				0.076 i
**>0–<7 hours/week**	13.1	1	23.6	1	8.35 (1.45)	0	18.5	1
≥7 hours/week	15.0	1.25 (0.72; 2.16)	30.1	1.35 (0.89; 2.05)	8.61 (1.63)	0.34 (−0.01; 0.69)	26.1	1.37 (0.94; 2.01)
	0.561	0.423	0.114	0.162	0.122	0.059	0.044	0.102
Continuous hours/week		0.583 i		0.912 i		0.03 (−0.003; 0.06)		0.142 i
		1.00 (0.95; 1.06)		1.01 (0.98; 1.05)		0.078		1.04 (1.00; 1.08)
		0.874		0.550				0.049
**Active smoking**		0.121 i		0.836 i				0.030 i
No	13.3	1	22.4	1	8.33 (1.43)	0	21.6	1
Yes	14.1	1.19 (0.53; 2.67)	50.8	2.65 (1.41; 4.99)	9.54 (2.14)	1.07 (0.39; 1.75)	4.5	0.29 (0.17; 0.51)
	0.871	0.669	<0.001	0.003	<0.001	0.002	0.001	<0.001
Ordinal coding for categories (never, <1 time/week, 1 time/week, several times/week, every day)		0.486 i		0.864 i		0.44 (0.23; 0.64)		0.020 i
		1.04 (0.81; 1.34)		1.54 (1.24; 1.90)		<0.001		0.66 (0.56; 0.79)
		0.741		<0.001				<0.001
**Passive smoking**		0.006 k		0.801 i				0.175 i
≤1 time/week	12.1	1	23.4	1	8.40 (1.61)	0	21.2	1
>1 time/week	16.9	1.41 (0.78; 2.54)	34.2	1.64 (1.02; 2.63)	8.75 (1.35)	0.15 (−0.22; 0.52)	13.6	0.74 (0.49; 1.13)
	0.158	0.251	0.016	0.040	0.064	0.419	0.058	0.162
Ordinal coding for categories (never, <1 time/week, 1 time/week, several times/week, every day)		0.028 i		0.839 i		0.13 (0.02; 0.24)		167 i
		1.23 (1.02; 1.49)		1.26 (1.08; 1.47)		0.017		0.89 (0.78; 1.02)
		0.034		0.004				0.099
**Television**		0.205 i		0.878 i				0.078 i
≤2 hours/day	13.9	1	23.0	1	8.41 (1.53)	0	21.3	1
≥3 hours/day	11.5	0.89 (0.45; 1.74)	35.2	1.03 (0.63; 1.68)	8.58 (1.66)	0.07 (−0.34; 0.49)	10.5	0.58 (0.37; 0.90)
	0.521	0.725	0.014	0.917	0.386	0.735	0.015	0.016
Ordinal coding for categories (never, 0.5 hours/day, 1–2 hours/day, 3–4 hours/day, ≥4 hours/day)		0.365 i		0.822 i		−0.09 (−0.26; 0.09)		0.078 i
		0.97 (0.73; 1.29)		0.94 (0.76; 1.16)		0.325		0.94 (0.78; 1.13)
		0.833		0.545				0.505
**Computer**		0.558 i		0.781 i				0.063 i
≤2 hours/day	13.6	1	20.6	1	8.32 (1.47)	0	21.8	1
≥3 hours/day	13.3	1.11 (0.63; 1.95)	39.4	1.62 (1.05; 2.51)	8.86 (1.76)	0.38 (0.00; 0.75)	13.2	0.59 (0.40; 0.87)
	0.930	0.731	<0.001	0.030	0.005	0.050	0.018	0.008
Ordinal coding for categories (never, 0.5 hours/day, 1–2 hours/day, 3–4 hours/day, ≥4 hours/day)		0.399 i		0.455 i		0.19 (0.02; 0.36)		0.072 i
		1.10 (0.87; 1.40)		1.23 (1.02; 1.48)		0.026		0.78 (0.66; 0.92)
		0.420		0.035				0.003

a Percentage of patients with respective outcome.

b P-value (χ^2^-Test).

c Odds ratio estimated from ordinal logistic regression (proportional odds model) adjusted for age, sex and socioeconomic status, separate model for each health related behaviour.

d P-value (Wald-χ^2^-Test).

e Mean Hba1c by health-related behaviour.

f P-value (t-Test).

g Difference estimated from linear regression adjusted for age, sex and socioeconomic status, separate model for each health related behaviour, ^d^p-value (t-Test).

h P-value (Wald-χ^2^-Test).

i P-value (Score test for proportional odds assumption).

j Physically inactive youths were excluded.

k As in this model the proportional odds assumption was rejected, we also estimated ORs in a non-proportional odds model. The OR estimates for ≥1 and ≥2 severe hypoglycaemic events were 1.34 (95% CI 0.74; 2.44, p = 0.340) and 3.19 (95% CI 1.34; 7.44, p = 0.020), respectively.

Youths with a higher frequency of physical activity had an increased odds for 1 and ≥2 severe hypoglycaemic events per year according to dichotomous (OR = 1.75, p = 0.026) and ordinal modelling of physical activity (OR 1.29, p = 0.045) (Table 5). There was no statistically significant association between hypoglycaemic events and overall weekly duration of physical activity, the use of different media, and active smoking. Passive smoking as dichotomous factor was not associated with hypoglycaemic events in the proportional odds model. However, the proportional odds assumption was questionable in this model. When specifically modelling non-proportional odds passive smoking modelled as binary variable was significantly associated with ≥2 hypoglycaemic events (OR 3.16, p = 0.009) but not with ≥1 severe hypoglycaemic events (OR1.34, p = 0.340). In ordinal modelling, passive smoking was associated with a higher odds for severe hypoglycaemic events (OR 1.23, p = 0.034) (Table 5).

Frequency and weekly duration of physical activity and longer use of television were not significantly associated with the level of HbA1c irrespective of binary or ordinal modelling of variables and according to both the ordinal logistic and the linear regression models. However, active smoking (dichotomous/ordinal modelling: OR 2.65/1.54, p = 0.003/<0.001), passive smoking (OR 1.64/1.26, p = 0.040/0.004), and longer computer use (OR 1.62/1.23, p = 0.030/0.035) were associated with higher odds for moderate and poor glycaemic control in ordinal logistic regression independent of binary or ordinal modelling. Likewise, these behaviours were associated with higher HbA1c levels according to linear regression with the exception of dichotomous passive smoking (Table 5).

Higher frequency of physical activity (dichotomous/ordinal modelling OR = 1.70/1.30, p = 0.002/0.002) was associated with a higher odds for at least good and excellent self-rated health status. However, longer weekly duration of physical activity showed as significant association with health status only when modelled continuously (OR = 1.04, p = 0.049). Active smoking (OR = 0.29, p<0.001) and more than three hours daily use of television (OR = 0.58, p = 0.016) and computer (OR = 0.59, p = 0.008) were associated with decreased odds for at least good and excellent self-rated health status. The association of active smoking and duration of computer use was confirmed when modelled in ordinal approach (OR 0.66, p<0.001 and OR 0.78, p = 0.003). Passive smoking showed no significant association with self-rated health-status.

Additional adjustment for insulin therapy regimen, diabetes duration, region of residence, weight status and HbA1c (not for HbA1c outcome) affected estimates only little (data not shown).

## Discussion

Children and adolescents with early disease onset and long diabetes duration are at particular risk for long term complications. Therefore, their health relevant behaviours have a high relevance. They differed from the peers of the general population in several aspects. A positive finding of our study is that children and adolescents with type 1 diabetes had a healthier profile with regard to smoking and time spent watching television compared to the general population in Germany. However, they were also characterized by less physical activity, and by spending more time at the computer.

The World Health Organization (WHO) recommends that youths should be physically active for at least one hour every day [15]. Only one third of children and adolescents of the KiGGS group was physically active as recommended. The children and adolescents of the diabetes study were physically active both less often and shorter than the reference group. An identical finding was reported very recently for youths with type 2 diabetes [25]. A reason may be the fear of hypoglycaemia associated with prolonged exercise [26]. Our explorative analyses support this hypothesis because frequency of physical activity was associated with a higher risk for severe hypoglycaemic events. Therefore, patient education should be intensified with regard to blood glucose awareness training, strategies to prevent activity-induced hypoglycaemia and behavioural therapy to manage the fear of hypoglycaemia.

Physical activity comes along with improved metabolic and cardiovascular parameters. There is an inverse association between physical activity performed regularly at least once a week for at least 30 minutes and HbA1c, and regular physical activity was not associated with a higher rate of severe hypoglycaemia based on a large patient database [2]. A review of 42 studies showed a significant relationship with insulin sensitivity in 78% of studies on physical activity, and 69% of studies on cardiorespiratory fitness [27]. Analysis of 23.251 patients from 209 centres in Germany and Austria revealed lower lipoprotein levels, diastolic blood pressure and HbA1c to be associated with increasing physical activity [28]. However, we could not confirm the beneficial effect of physical activity on HbA1c. Results indicated that longer overall physical activity per week was associated with a lower chance for optimal HbA1c and a higher risk for poor HbA1c. Also other authors raise questions about the beneficial effects of physical activity on metabolic control, using a similar measure for physical activity [29]. Perhaps, the used methods for assessing physical activity are not sensitive enough to detect an association.

A positive association was observed between physical activity and excellent self-rated health. This is in accordance with other studies, in which physical activity was positively correlated with nearly all markers of psychological health, including well-being, less worry, greater perception of health and general quality of life in patients with type 1 diabetes [29], [30] as well as healthy peers [31]. However, most studies may not show the causal direction of the association between physical activity and self-rated health, as both influence each other.

In studies among older adolescents and emerging adults the observed prevalence of smoking was usually similar with and without diabetes [7], [32], [33]. In our study, the prevalence of regular active and passive smoking was only half as high as in the general population. This finding might be to some extend attributable to a cohort effect arising from the time span of 3-7 years between the data collection in both studies. Since the beginning of this millennium, a significant decrease in smoking prevalence was observed in the general population, as shown for adolescents in the USA [34] and Germany (decrease from 20.4% to 12.0 % between 2003-6 and 2009-12) [35]. However, children and adolescents with other chronic diseases, such as sickle cell anaemia and cystic fibrosis, reported less frequent use of tobacco than healthy controls as well [36]. This may indicate a generally better health awareness of the youths with type 1 diabetes – like for other chronic diseases - with regard to active smoking, because it is part of the education program for patients [37]. The reduced passive tobacco exposure might be caused by the youths' active avoidance of tobacco smoke or due to an improved health awareness of their family and friends. It was not explained by socioeconomic status or migration background, as analyses were adjusted for both variables. Smoking during adolescence increases the risk for developing chronic complications in patients with type 1 diabetes [38]. A more recent observation was that tobacco exposure is associated with worse glycaemic control and markers of higher cardiovascular risk already in children and adolescents with type 1 diabetes [39]. Our results confirm that active smoking is associated with an increased odds for poor glycaemic control in youths with early-onset type 1 diabetes. Moreover, smoking was associated with a decreased odds for excellent self-rated health status in our study population. This may be due to direct adverse effects of tobacco exposition or may reflect a generally lower health-promoting lifestyle in patients who smoke.

Patients showed increased computer use and decreased time spent watching television compared to KiGGS participants, which could at least in part be due to earlier data collection. During the past years, computers and internet became more available to youths. This cohort effect might explain why markedly less children and adolescents of the diabetes study than of the KiGGS reported to use a computer “not at all”. The difference in time spent watching television is probably not due to time effects, because available literature suggests a minimal increase in daily television use over time [40]. Negative impact of media consumption on health is well documented in children and adolescents, as it increases the likelihood of overweight, unhealthy food preferences, and smoking [41]. Furthermore, it reduces beneficial activities such as reading or hobbies with physical activity [41]. In children and adolescents with type 1 diabetes, an increased daily time of media consumption is associated with higher HbA1c [42]. These findings are confirmed by our population-based study as both increased computer use and increased television consumption were associated with poor self-reported HbA1c. The association between less media consumption and optimal HbA1c was less pronounced. A new finding was that media consumption was negatively associated with self-rated health status. Possibly, children and adolescents with T1DM feeling more impaired have fewer joint activities with friends and prefer physically inactive leisure time activities. This behaviour might reduce their self-rated health and their motivation to be physically active (vicious circle).

A limitation of this study is that data, in particular on health-related behaviours, are based on self-administered questionnaires. These tend to over-estimate behaviours that participants believe to be socially desirable and underestimate behaviours that are undesirable. This limitation is unavoidable in population-based studies evaluating these factors, and can be assumed to be non-differential between both study groups. In addition, children and adolescents of the diabetes study were not as representative as the KiGGS participants were. However, as both groups were evaluated using identical questionnaires and confounding factors were considered in the analyses, it is unlikely that the observed between-group differences were artefacts due to differential reporting bias. The possible cohort effect with respect to computer use has already been mentioned. A limitation of the explorative analyses of the effects of health related behaviours on outcomes is the cross-sectional study design that hampers the differentiation between causes and consequences, e.g. it is possible that general health status has an impact on physical activity and vice versa. Further, HbA1c values from clinical records were available only for about 71% of patients limiting the analyses with respect to HbA1c to this subgroup.

In summary, we revealed that children and adolescents with early-onset and long-duration type 1 diabetes tended to smoke less, watch fewer TV, use the computer more often and be less physically active than the general population. Our study provides further evidence that less physical activity, more frequent media use and smoking are associated with worse glycaemic control and worse self-rated health in children and adolescents with type 1 diabetes. There is still the need for reduction of activity-related hypoglycaemia, while patients should be further encouraged to increase their overall physical activity and to decrease sedentary behaviours in order to achieve positive effects on health-status, glycaemic control and long-term sequelae of diabetes. In conclusion, besides physical activity and smoking, media consumption is an important factor contributing to the overall health and glycaemic control in children and adolescents with type 1 diabetes and should be intensely considered in diabetes education.
